# 

**Published:** 1879-06

**Authors:** 


### ARTICLE VII.

### Dental Associations.

### *National Dental Association.*

The Eleventh Annual Meeting of this Association, (late "Southern,") will be held in Augusta, Georgia, commencing on the 2nd Tuesday in July, 1879, at 10 A. M.

It is expected that the members of the Georgia, South Carolina and North Carolina State Dental Societies will be present, and a cordial invitation is extended to the profession generally.

Officers.

*President.*—Prof. F. J. S. Gorgas, Baltimore, Md.

1*st Vice President.*—Dr. L. D. Carpenter, Atlanta, Ga.

*2nd Vice President.*—Dr. J. R. Walker, New Orleans, La.
*3rd Vice President.*—Dr. John G. Wayt, Richmond, Va.
*Cor. Secretary.*—Dr. A. C. Ford, Atlanta, Ga.
*Rec. Secretary.*—Dr. E. S. Chisholm, Tuscaloosa, Ala.
*Treasurer.*—Dr. H. A. Lowrance, Athens, Ga.
*Executive Committee.*—Drs. W. C. Wardlaw, G. H. Winkler, of Augusta; G. W. H. Whitaker, Sandersville, Ga.

The members of the different Standing Committees are earnestly requested to be present, and present papers on the subjects designated. Proper arrangements will be made for reduction of Rail Road fares, &c., by the Executive Committee.

E. S. Chisholm,
*Recording Secretary.*

---

### *South Carolina State Dental Association.*

The Ninth Annual Meeting of the South Carolina State Dental Association will be held in the office of the President, J. B. Patrick, D. D. S., July 7th, 1879, at 10 A. M., in Charleston, S. C. That afternoon the Association will adjourn to meet in joint session with the Southern Dental Association and Georgia Dental Society, next morning, July 8th, at Augusta, Ga.

All respectable dentists are informed, that it is their privilege and duty to attend one or both of these meetings. Every inducement which can, will be afforded the profession.

The State Board of Dental Examiners will meet at Charleston, in the office of Dr. J. B. Patrick, July 7th, 1879, at 10 A. M.

Candidates for the practice of dentistry in South Carolina must apply, at or before that time, to Dr. W. S. Brown, of Charleston.

The attention of graduates in dentistry subsequent to February 23, 1875, is called to Sections 3rd and 7th of an Act of the General Assembly, to regulate the practice of dentistry in this State.

G. F. S. Wright.
*Rec. Secretary, Columbia S. C.*

*Georgia State Dental Society.*

The Eleventh Annual Session of the Georgia State Dental Society will be held in the city of Augusta, Ga., on Tuesday, the 8th of July next, at 10 o'clock A. M.

The State Board of Dental examiners meets at the same time and place.

L. D. Carpenter,
*Cor. Secretary.*

---

### EDITORIAL, ETC.

### To Readers and Correspondents.

Communications intended for publication, and exchange journals and papers, should be addressed to the Editor of the American Journal of Dental Science, 259 N. Eutaw St., Baltimore, Md. All letters relating to the business of the journal, or containing cash remittances, to be directed to Messrs. Snowden & Cowman. Articles for publication should be received by the editor at least three weeks previous to the first day of the month on which the number in which it is designed for them to appear, is issued.

### MONTHLY SUMMARY.

Rapid Anæsthesia .......... 93
Mal-Practice Case .......... 94
Chewing Gum .......... 95
A Remarkable Old Man .......... 95
Chloroform Accidents .......... 96
How to Care for the Eyes .......... 96
Action of Substances on the Teeth .......... 96

☞ The American Journal of Dental Science will contain fifty pages of reading matter in each number, making a volume of about

*Six Hundred* pages

EXCLUSIVE OF ADVERTISEMENTS

THE

### AMERICAN JOURNAL

OF

### DENTAL SCIENCE.

The *Journal* is issued on the first of each month and contains not less than *fifty* pages of reading matter in each number, exclusive of advertisements, making a volume of *six hundred* pages per annum.

In each number will be found Original Communications, Correspondence, Selected Articles, Monthly Summary, Bibliographical Notices and Editorial Matter, and every effort will be exerted to render the *Journal* worthy of the patronage of the Dental profession.

A generous co-operation is respectfully solicited from the members of the profession; and as the *Journal* has now a printing office of its own, which materially lessens the cost of its publication, it will be issued at the reduced subscription price of

**$2.50 Per Annum in Advance.**

Communications are respectfully solicited on all subjects pertaining to the practice of dentistry, as well as reports of cases occurring in dental practice.

### RATES OF ADVERTISING.

| | 1 MO. | 2 MO. | 3 MO. | 6 MO. | 12 MO. |
|---|---|---|---|---|---|
| 1 PAGE. | $15 00 | $22 50 | $30 00 | $50 00 | $100 00 |
| ½ " | 10 00 | 15 00 | 20 00 | 30 00 | 50 00 |
| ¼ " | 7 00 | 11 00 | 15 00 | 20 00 | 30 00 |
| ⅛ " | 5 00 | 8 00 | 11 00 | 15 00 | 20 00 |

All original communications designed for publication, works for review, new instruments for notice, exchanges, &c., should be directed to the editor, 259 N. Eutaw St., Baltimore, Md.

All advertisements and subscriptions should be addressed to

SNOWDEN & COWMAN,

*Publishers of the Am. Journal of Dental Science.*

86 W. FAYETTE ST. BET. CHARLES & LIBERTY STS.,

BALTIMORE, MD.

